# A pilot feasibility study investigating the impact of increasing sucrose intakes on body composition and blood pressure

**DOI:** 10.1017/jns.2021.55

**Published:** 2021-08-11

**Authors:** Sophie Scott, Julie Young, John K. Lodge

**Affiliations:** Department of Applied Sciences, Northumbria University, Ellison Building, Newcastle-Upon-Tyne NE3 7ST, UK

**Keywords:** Body composition, Blood pressure, Human intervention, Sucrose

## Abstract

Epidemiological and intervention studies have reported negative health effects of sucrose intake, but many of these studies were not representative of typical dietary habits. In this pilot study, we aimed to test the effect of increasing sucrose intakes for 1 week on body composition and blood pressure and explore the feasibility of consuming high intakes of sucrose in addition to a habitual diet. In a randomised crossover design study, twelve healthy participants (50 % female, age 28⋅4 ± 10 years, BMI 25 ± 3 kg/m^2^), consumed either 40, 80 or 120 g sucrose in 500 ml water in addition to their habitual diet every day for 1 week, with a 1-week washout between treatment periods. Body composition (assessed using bioelectrical impedance) and blood pressure measurements were taken before and after each intervention phase. All participants reported no issues with consuming the sucrose dose for the intervention period. There was a significant increase in systolic blood pressure following 120 g sucrose intake (*P* = 0⋅006), however there was no significant changes to blood pressure, body weight, BMI, percentage protein, fat or water (*P* > 0⋅05) when comparing change from baseline values. There was also no effect of sucrose intakes on energy or macronutrient intakes during the intervention (*P* > 0⋅05). We show here that varying doses of sucrose over a 1-week period have no effect on body composition or blood pressure. The amounts of sucrose used were an acceptable addition to the habitual diet and demonstrate the feasibility of larger-scale studies of chronic sucrose supplementation.

## Introduction

Sucrose is a disaccharide comprised of the monosaccharides glucose and fructose and is found widely within foods such as fruits and vegetables, confectionary and beverages. Sucrose from certain food sources can also be classified as a free sugar which nutritionally is defined as ‘all added sugars in any form; all sugars naturally present in fruit and vegetable juices, purées and pastes and similar products in which the structure has been broken down; all sugars in drinks (except for dairy-based drinks)’^(1)^. UK recommendations presently state that intake of free sugars should be no greater than 5 % of total daily energy^(2)^, equivalent to approximately 30 g/d in adults. Efforts to convert more of the population into understanding these recommendations are very much at the forefront of public health interventions as the present UK average consumption of free sugars for children (11–18 years) is 14⋅1 and 11⋅1 % for adults^(3)^. With free sugars providing an increase in caloric intake/energy density, there is an increased risk of developing obesity and non-communicable diseases, including cardiovascular disease and type 2 diabetes mellitus^(2,4)^. Attempts to reduce increases in obesity have included the introduction of policies focusing on consumption of free sugars, such as a sugar levy on drinks containing more than 8 g of sugar per 100 ml. Food manufacturers have also been challenged with reducing sugar content by 20 % across ten categories of food products including cakes, breakfast cereals, yoghurts and confectionary^(5)^.

Sucrose, or the individual monosaccharides, have been investigated for their metabolic effects in humans. For example, a significant dose-response effect of high-fructose corn syrup (HFCS) was found with circulating lipid and lipoprotein risk factors in young adults^(6)^, however no effects on measures related to glycaemic control were found in diabetic subjects^(7)^. There have been several intervention studies using sucrose on end points associated with anthropometric measures and clinical parameters, but results have been contradictory. Comparing a 5 % *v.* 15 % sucrose intake in an equicaloric diet for 6 weeks showed no difference to weight, body composition or insulin resistance^(8)^. Body weight was, however, increased in a study comparing sucrose-sweetened beverage intake (2 kg/kg body weight) *v.* artificial sweeteners for 10 weeks^(9)^. Interestingly, sucrose or fructose (at 25 % energy for 2 weeks) had differing effects on leptin even without a change to body weight^(10)^. A meta-analysis of randomised controlled intervention trials with sucrose^(11)^ has found no influence of sucrose on weight gain even though evidence suggests that sugar consumption is associated with excess energy and may predispose to weight gain and adiposity^(4,12)^. Weight gain appears to be a driving force for other metabolic changes as sucrose intakes have been associated with lipid status in some intervention studies^(6)^, but not in others where there was no weight gain^(13)^. There are several studies that indicate a relationship between sugar intake and blood pressure^(14,15)^. The Framingham Heart Study reported an association with consuming >1 sugar-sweetened beverages per day on high blood pressure^(15)^, whereas randomised controlled trials have found mixed results.

Many of the above studies report contradictory findings and are of different designs with durations of 2 weeks to several months, and with studies designed to control for total energy intake which may not be representative of habitual intakes. Understanding the acceptability of sucrose consumption is key for planning future interventions, and the aim of the present pilot study, therefore, was to test the feasibility of chronic sucrose intakes on selected health parameters to inform the design of larger intervention studies.

## Methodology

### Materials

25 kg of caster sugar (99⋅9 % sucrose) was purchased from Tate and Lyle. Participants were provided with a 600 ml plastic drink receptacle (MX^©^ shaker bottle).

### Transparency declaration

Wherever possible we have complied with the CONSORT guidelines for the reporting of randomised trials. The study was approved by Northumbria University Faculty Ethics Committee and has been conducted according to the principles expressed in the Declaration of Helsinki. The study has been registered with ClinicalTrials.gov (identifier NCT04486105).

### Study design and protocol

The study was a randomised crossover design, where all participants supplemented their habitual diet with 40, 80 and 120 g of sucrose for 1 week with a 1-week out period between doses (Supplementary Fig. S1). Randomisation was performed using web-based software (https://www.randomizer.org). Participants completed an initial questionnaire about present dietary habits and lifestyles that determined their eligibility for the study. Following recruitment and randomisation, participants completed a validated weighed 3-d food diary^(16)^, and attended a baseline study visit where they received their allotted sucrose supplements for the whole duration of the study, together with instructions on how the sucrose should be consumed. Participants were asked to not make any changes to their habitual diet or physical activity throughout the study and were advised to consume the sucrose dissolved in water and consume in three sittings throughout the day or with meals. They were asked to return any unused sucrose on their next visit. Participants then had their blood pressure, height, weight and body composition measured. Participants were asked to maintain their habitual diet throughout the duration of the study. During the intervention period, participants recorded three 1-d food diaries on days 2, 4 and 6. Following the intervention period, participants attended Northumbria University and measurements were recorded as for the baseline day. All measurements were performed by the same researcher (S. S.). On completion of the study, participants were asked about compliance to the intervention (and return of any unused sucrose) as part of a ‘post-study questionnaire’. Participants were asked questions about any difficulties consuming the dissolved sucrose, did they consume the entire dose and how, and for how many days they adhered to the intervention. Participants were also asked to complete an ‘acceptability questionnaire’ regarding their experiences throughout each week of the intervention. Participants were asked questions about their own perceived carbohydrate/sucrose consumption, and if they possessed any knowledge/awareness of present government guidelines on sugar consumption.

### Participants

Although this was a pilot study designed to inform on feasibility for a larger intervention, we performed a sample size calculation based on changes to BMI. Using the mean BMI (26⋅12 kg/m^2^ ± 5⋅92) of UK adults taken from the National Diet & Nutrition Survey (NDNS) UK rolling programme, to detect a difference of 6 kg/m^2^ (equivalent to sd) in BMI as our primary end point at 80 % power would require sixteen individuals. Inclusion criteria were healthy males and females aged 18–65 years old located in the Newcastle Upon Tyne area. Participants were excluded based on the following criteria, if they have been diagnosed and undergoing treatment for any pre-existing medical conditions/illnesses such as type 1 or type 2 diabetes mellitus or any other metabolic complications, have any blood disorders or know of any infections, have a history of significant head trauma or suffer from frequent migraines that require medication (more than or equal to 1 per month), have a visual impairment that cannot be corrected with glasses or contact lenses are pregnant, trying to get pregnant or breast feeding, have donated more than 300 ml of blood in the past 3 months, are presently taking any prescription medication or have regularly used dietary/herbal supplements within the last month (defined as 3 consecutive days or 4 d in total), have a BMI of >29⋅9 kg/m^2^ or have any conditions which would inhibit fulfilment of the study requirements. Recruitment occurred via social media and resulted in a total of twelve people volunteering. All provided their written informed consent. Recruitment proceeded from June to October 2019.

### Blood pressure

Blood pressure was recorded using an OMRON Automatic Blood Pressure Monitor-M6 Comfort model (Japan); three measurements were taken in the sitting position at rest and averaged. Accuracy of the device has been determined previously^(17)^.

### Body composition measures

Body weight, BMI, % body muscle, % body fat and % body water were recorded with a Tanita BC-418 MA bioelectrical impedance monitor. Measurements were taken at consistent times and participants were asked to consume the same food and drink prior to each visit. Accuracy has been determined previously^(18)^. Values are expressed as a percentage so that differences were relative to each participant. Height was recorded using a wall-mounted SECA stadiometer.

### Dietary analysis

Clarity of the food diary entries was checked by the researcher (S. S.). Food diaries were converted to nutrient intakes using Nutritics Professional Plus 2020, nutritional analysis software. Data were then exported to Excel and SPSS for further manipulation and analysis. Only total energy (kcal/d) and macronutrient intake (g/d) data were used.

### Statistical analysis

Data were tested for normality using the Kolmgorov–Smirnov test, and then comparisons of means tests for dependent samples were used. Within treatment differences (pre *v.* post) were analysed by either paired *t*-tests or a Wilcoxin signed-rank test. Between treatment effects for anthropometric, blood pressure and dietary data were analysed by a general linear model (GLM) with log-transformed data if nonparametric, using treatment (sucrose dose) and gender as fixed factors with covariates age and energy intake. Significance was considered at the *P* < 0⋅05 level. All analyses were performed using IBM SPSS version 24.

## Results

Demographic and baseline data for the study participants are shown in Table 1. The participants had a mean age of 30⋅2 ± 10 years with six males and six females. All participants were in the healthy range for blood pressure and anthropometric measures, however although three participants were in the overweight category for BMI, they met the inclusion criteria for the study. Height was consistent at 166⋅8 ± 8 cm. There were no significant differences between these measures at baseline (*P* > 0⋅05). All participants consumed all sucrose doses throughout the intervention (100 % compliance) and also self-assessed no ill effects.

**Table 1.** Baseline characteristics of the study population (*n* 12) at each intervention stage

Characteristic	Baseline	Pre 40 g	Pre 80 g	Pre 120 g
Systolic blood pressure	121⋅5 ± 0⋅9	120⋅8 ± 5⋅8	121⋅1 ± 4⋅9	122⋅5 ± 5⋅2
Diastolic blood pressure	73⋅3 ± 2⋅1	73⋅6 ± 12⋅7	75⋅3 ± 9⋅7	71⋅1 ± 6⋅1
Weight (kg)	70⋅3 ± 0⋅3	70⋅3 ± 10⋅8	70⋅5 ± 10⋅9	70⋅0 ± 10⋅9
BMI (kg/m^2^)	25⋅2 ± 3⋅1	25⋅3 ± 3⋅2	25⋅3 ± 3⋅2	25⋅1 ± 3⋅2
Body fat (%)	21⋅9 ± 0⋅1	21⋅9 ± 6⋅7	21⋅8 ± 6⋅5	21⋅9 ± 6⋅3
Body muscle (%)	33⋅0 ± 0⋅1	32⋅9 ± 4⋅1	32⋅9 ± 3⋅9	33⋅1 ± 4⋅4
Body water (%)	61⋅8 ± 0⋅3	61⋅6 ± 5⋅3	61⋅8 ± 5⋅3	62⋅1 ± 2⋅6

No significant differences between the treatment conditions were detected.

**Fig. 1.**
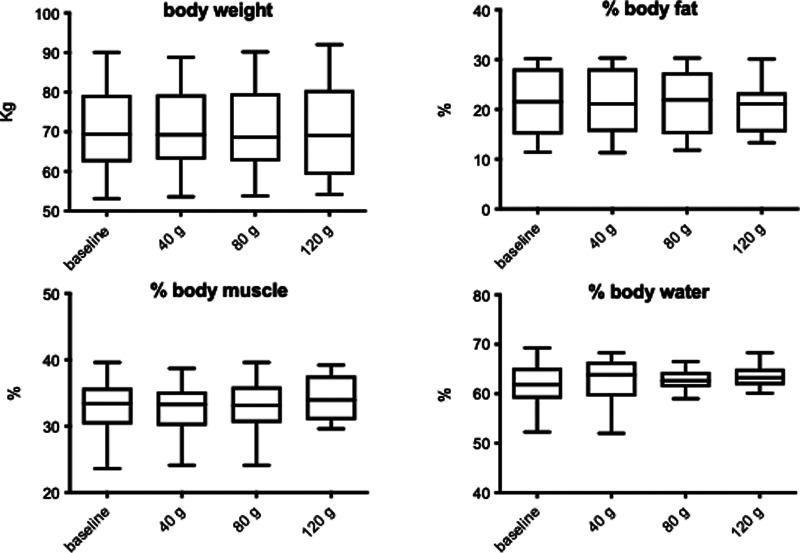
The influence of increasing sucrose intake on anthropometric measurements. Data shown are means ± sd.

The influence of increasing doses of sucrose on body composition is shown in Fig. 1. There was no significant change to weight (*F* 0⋅003; *P* = 0⋅99), % body fat (*F* 0⋅91; *P* = 0⋅445), % body water (*F* 0⋅53; *P* = 0⋅665) or % body muscle (*F* 0⋅55; *P* = 0⋅651), although % body muscle did show a slight increase following the 120 g sucrose intervention. There was also no significant change to BMI (*F* 0⋅004; *P* = 0⋅99 data not shown). Some of these parameters were significantly changed when analysing pre- *v.* post-treatments. There was a significant decrease in % body fat (*P* = 0⋅037), and a significant increase in % body muscle (*P* = 0⋅043) following 120 g sucrose. No gender interactions were found.

**Fig. 2.**
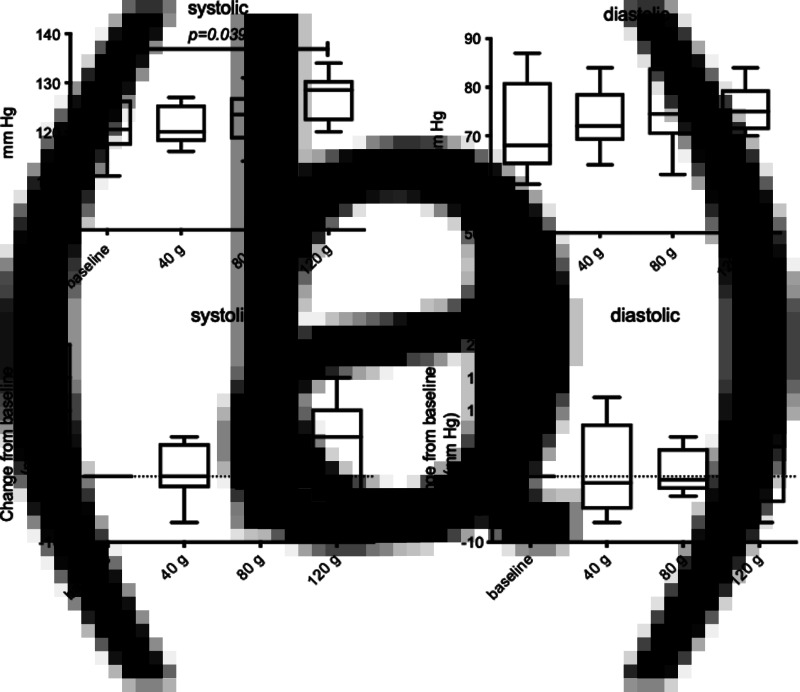
The influence of increasing sucrose intake on blood pressure measurements. Actual values shown in (a), change from baseline values are shown in (b). All values are mean ± sd, there was a significant increase in systolic blood pressure with sucrose treatment (*P* = 0⋅006), and also comparing pre *v.* post 120 g sucrose (*P* = 0⋅039).

**Fig. 3.**
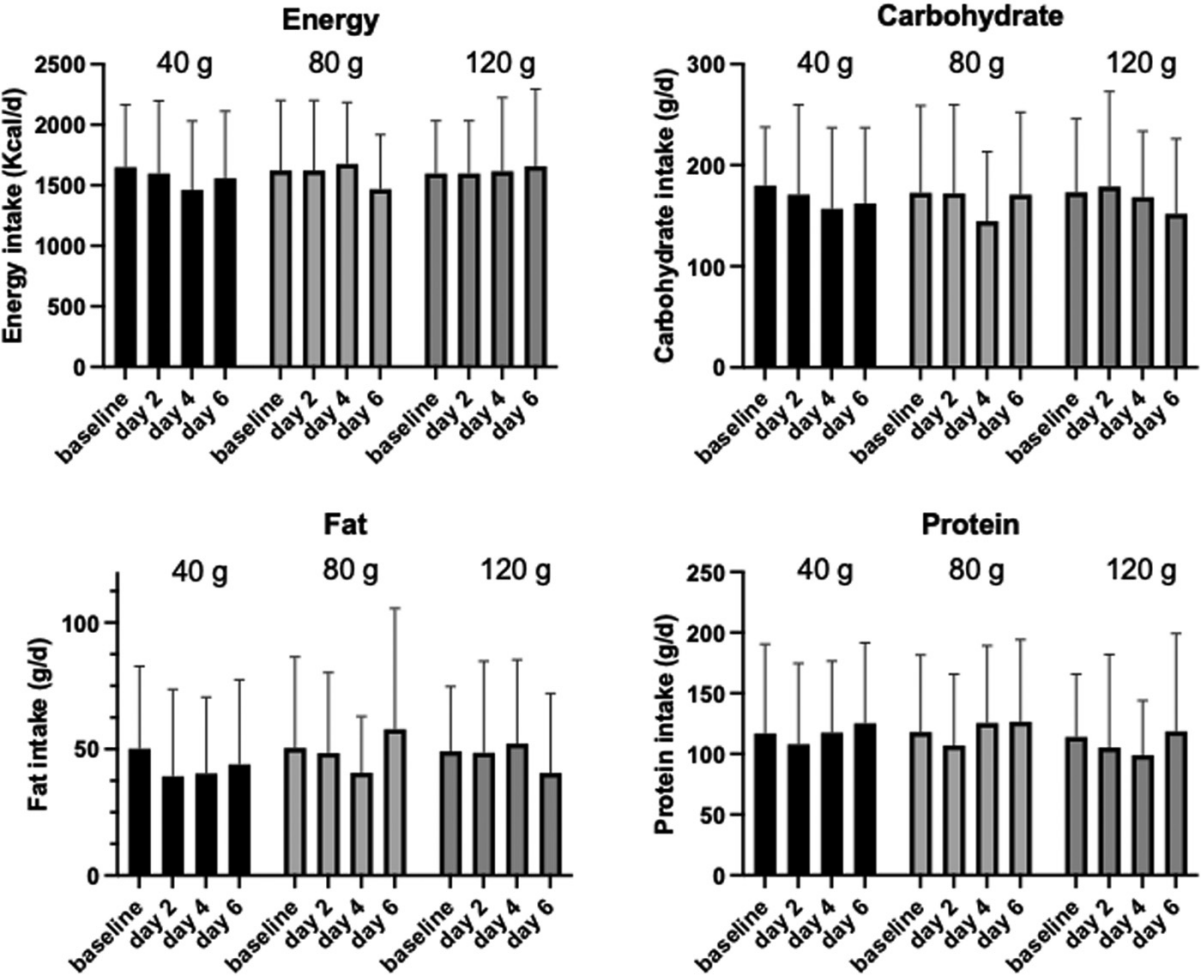
Nutritional intake over the course of the intervention. Values are mean ± sd. No significant differences were found.

The influence of increasing doses of sucrose on blood pressure is shown in Fig. 2. There was a significant increase in systolic (*F* 4⋅74; *P* = 0⋅006), but no change to diastolic (*F* 1⋅40; *P* = 0⋅256) blood pressure. Systolic blood pressure was increased in a dose-dependent fashion from 120 to 127 mmHg (*P* = 0⋅006; Fig. 3(a)). There was no interaction with gender or sucrose treatment*gender. When analysing pre- *v.* post-treatments, there was a significant increase in systolic blood pressure following 120 g (*P* = 0⋅039). When the data are shown as change from baseline (Fig. 2(b)), this constituted a 7 mmHg increase, but this was not significant, although there was a trend for systolic blood pressure to increase with increasing sucrose dose (*F* 2⋅54; *P* = 0⋅079). At any stage of the intervention, blood pressure did not increase into the hypertensive zone.

Any dietary change focusing on energy and macronutrients was monitored through 1-d food diaries taken on days 2, 4 and 6 of the intervention and compared to a 3-d averaged food diary taken at baseline. Overall, there was no significant change to either energy or the macronutrients over the intervention period (Fig. 3; *P* > 0⋅5), although there was a significant gender interaction with energy intake following 40 g sucrose treatment (*P* = 0⋅024) and with protein intake following all treatments (*P* < 0⋅01). Dietary factors that could compound findings (e.g. caffeine intake) were also investigated, however, there were no significant differences between pre- and post-intervention.

Questionnaire data assessing acceptability of supplemented sucrose intake and general nutrition knowledge showed that 58 % of participants were not knowledgeable regarding government carbohydrate consumption guidelines. Also, 83 % of individuals were able to provide a value for their perceived, personal carbohydrate consumption and only 75 % were able to provide a value for their sugar consumption. On analysis of perceived carbohydrate consumption in comparison to actual carbohydrate consumption, the mean average inaccuracy was underestimated by 9⋅8 ± 8⋅5 %.

## Discussion

This pilot study has shown that increasing doses of sucrose up to 120 g/d for 1 week, in addition to the habitual diet, does not change any measure of body composition or changes to energy and macronutrient intake, but did cause changes to blood pressure in an adult population. The present study is the first of its kind, intervening with increasing doses of sucrose for 1 week and monitoring body composition and blood pressure following each stage.

We found a significant increase in systolic blood pressure following 120 g sucrose per day, although significance was lost when values were displayed as change from baseline. There are several lines of evidence indicating a relationship between sucrose intake^(14,15)^ or fructose intake^(19)^, and blood pressure, however these studies are observational and there is limited evidence from interventions trials. In one randomised controlled trial with large doses of fructose (200 g/d for 2 weeks), there was a significant increase to both systolic and diastolic blood pressure^(20)^, and the authors suggest that this effect may be due to the increase in renal inflammation induced by serum uric acid^(19,20)^. However, fructose containing sugars at normal levels of intake for 10 weeks^(21)^ or up to the 90th percentile level (30 % of calories) for 10 weeks^(22)^ did not influence blood pressure. Acute increases in systolic blood pressure have been reported in association with consumption of pure sucrose, following supplementation of 1 g of sucrose per kg of body weight, systolic blood pressure increased by 9 mmHg on average after 1 h^(23)^; but as the effects observed occurred across a 24 h period, it is difficult to determine if these increases are transient or remain elevated following completion of the study. The Framingham Heart Study reported an association with consuming >1/d sugar-sweetened beverage and high blood pressure^(15)^, and previously, it has been shown that consumption upwards of 1 l of sugar-sweetened beverages (actual quantities of sucrose were not provided) have an association with slight increases in systolic blood pressure, with a *z*-score increase of 0⋅17^(24)^. Since this study did not report which sugar-sweetened beverages were consumed, it is unclear what volume of sucrose was ingested and what caused the increase in systolic blood pressure^(24)^. A further explanation for the increase in systolic blood pressure could be a change in other dietary factors, although we found no changes to macronutrient, micronutrient or caffeine intake. Whatever the exact mechanism of effect, any changes to blood pressure induced by chronic sucrose intake could translate to more chronic changes and increased risk of cardiovascular diseases as suggested by SACN^(2)^.

We did not see any sucrose dose-dependent changes to body weight and % body fat in our study. A large amount of research evidence from systematic reviews suggests that sugar consumption is associated with weight gain and adiposity in epidemiological studies^(4,25)^. Recently, a study with a similar supplementation period of 2 weeks but with glucose and HFCS, found significant increases in body weight in young adults^(10)^. Contrary to this, studies that have assessed the effects of increased sucrose intake have observed no significant differences in body weight or body composition over 4 weeks^(8,26)^. There have also been studies investigating fat mass changes with sucrose intake over longer durations. During a 6-month randomised intervention, participants were provided with either a sucrose-sweetened beverage, an isocaloric milk drink or a noncaloric soft drink and although no significant differences in total fat mass were observed, significant increases in liver, visceral and intramuscular fat were seen^(27)^. Similarly, fructose supplementation at 25 % of daily energy intake for 10 weeks resulted in approximately 3 % increase in body fat, 8 % increase in abdominal fat and a 14 % visceral fat in humans^(28)^. This is of particular interest as fat deposition can lead to metabolic dysregulation, and further risks of insulin resistance^(29)^.

Following the 120 g sucrose supplement, small increases in % body muscle were observed in several participants that were statistically significant with this treatment dose only. One potential reason for this could be an increase in glycogen. Skeletal muscle glycogen contributes 1–2 % of total body weight^(30)^ and with participants being asked to consume an additional 120 g of carbohydrates a day, it is plausible that muscle glycogen stores may have been fully saturated, potentially leading to this observed increase in body muscle. Alongside glycogen storage, it is said that 3 ml of water is also stored alongside every gram of glycogen^(31)^, so an increase in intramuscular water is likely to be recorded when using bioelectrical impedance.

We have also shown here, albeit in a pilot study of a small population, the acceptability of increasing sucrose intake to 120 g/d in addition to the habitual intake, making this plausible for a nutritional intervention on a larger scale. As showcased in the NDNS rolling programme, the non-low calorie soft drink and sugar-sweetened beverages category are the major sources of sucrose intake in UK adults^(2)^. Mean sucrose consumption is 41⋅5 ± 26⋅3 g/d, ranging between 0⋅49 and 219 g/d, with intakes of non-low calorie soft drinks consumption at 94 ± 197⋅5 ml. This value of intake is similar to that reported by the British Soft Drinks Association (2019), where consumption in 2018 of carbonated soft drinks was on average 220 ml/d per person; 45⋅5 % of which was non-low calorie drinks giving an average daily consumption of 100 ml. The highest dose used in the present study (120 g/d) falls well within this range. With this in mind, in order to fulfil the upper amount of sucrose supplemented, as an example approximately 4⋅2 cans of Fanta®, or 2⋅8 cans of Pepsi® would need to be consumed on a daily basis.

The present study is not without limitations, although some significant differences in blood pressure were observed, a larger sample size may have demonstrated lower variation with more confidence of observing significant results. The present study was only 1-week duration when longer time periods may be required to see changes in the end points. Participant activity, hydration levels and daily energy expenditure were not measured or controlled throughout the study and although participants did not report large variation in normal exercise or dietary patterns, it is a potential source of error and can influence anthropometric assessment. Such limitations will be important to take forward with larger-scale sucrose intervention studies with a more controlled diet, hydration and physical activity with adequate control in order to deduce the sole effects of sucrose.

## Conclusions

We have shown here in this pilot study that chronic sucrose intakes at dosages of 120 g/d for 1 week significantly increased systolic blood pressure but no change to body composition, energy or macronutrient intake. The present study successfully demonstrated the feasibility and acceptability of increasing sucrose intake to this level on top of habitual diet with 100 % adherence to the intervention, with no reported side effects or changes to the diet. Therefore, larger supplementation studies of chronic sucrose supplementation should be feasible.

## Supplementary material

For supplementary material accompanying this paper visit http://dx.doi.org/10.1017/jns.2021.55. click here to view supplementary material
