# Supplementary material for: A pilot feasibility study investigating the impact of increasing sucrose intakes on body composition and blood pressure
Source: J Nutr Sci. 2021 Aug 11;10:e60. doi: 10.1017/jns.2021.55 (PMC8358843; doi:10.1017/jns.2021.55)
Supplement: Supplementary file 1 [file S2048679021000550sup001.doc]

**CONSORT 2010 Flow Diagram**

**Allocation**

**Analysis**

**Follow-Up**

**Enrollment**

Assessed for eligibility (n=12)

Excluded (n=0)

♦  Not meeting inclusion criteria (n=0)

♦  Declined to participate (n=0)

♦  Other reasons (n=0)

Analysed (n=12)
♦ Excluded from analysis (n=0)

Lost to follow-up (n=0)

Discontinued intervention (n=0)

40 g sucrose

Allocated to intervention (n=12)

♦ Received allocated intervention (n=12)

Randomized (n=12)

Analysed (n=12)
♦ Excluded from analysis (n=0)

Lost to follow-up (n=0)

Discontinued intervention (n=0)

120 g sucrose

Allocated to intervention (n=12)

♦ Received allocated intervention (n=12)

Analysed (n=12)
♦ Excluded from analysis (n=0)

Lost to follow-up (n=0)

Discontinued intervention (n=0)

80 g sucrose

Allocated to intervention (n=12)

♦ Received allocated intervention (n=12)
